# Lipid Deterioration Mitigation in Brown Rice Milled from Long-Term Stored Paddy by Microwave: A Lipidomic Perspective

**DOI:** 10.3390/biom16030419

**Published:** 2026-03-12

**Authors:** Senfan Luo, Beibei He, Li Wang, Luyao Zhao, Weiwei Wang

**Affiliations:** 1 School of Health Science and Engineering, University of Shanghai for Science and Technology, Shanghai 200093, China; lsflwnbz@126.com; 2 Key Laboratory of Grain and Oil Biotechnology, Academy of National Food and Strategic Reserves Administration, Beijing 100037, China; hbb@ags.ac.cn (B.H.); wl@ags.ac.cn (L.W.)

**Keywords:** brown rice, long-term stored paddy, microwave treatment, free fatty acid value, lipidomics, synergistic degradation

## Abstract

The utilization of brown rice from long-term stored paddy is severely limited by lipid deterioration, which is primarily characterized by a high free fatty acid value (FAV). Although microwave treatment shows promise in mitigating lipid deterioration, its underlying mechanism in degraded grains remains poorly understood. This study systematically investigated the efficacy and mechanism of microwave treatment using a multi-analytical approach. Brown rice from long-term stored paddy (*Longjing-46*, stored for 6 years) was treated using a laboratory microwave oven (420 W or 560 W, 1–5 min). The reduction in FAV was quantified, lipid structural changes were analyzed by FT-IR spectroscopy, and lipid metabolic alterations were profiled using untargeted lipidomics. Results showed that microwave treatment significantly reduced FAV in a time- and power-dependent manner, with a maximum reduction of 76.3%. Treatment at 420 W for 4 min was identified as the optimal condition. FT-IR analysis confirmed that the treatment inhibited lipid oxidation and hydrolysis at the molecular level. Importantly, lipidomics revealed that the mechanism extended beyond simple enzyme inactivation. Microwave treatment induced a reprogramming of the lipid metabolic network, characterized by the synergistic downregulation of key lipid species and the activation of the autophagy pathway. This study provides a comprehensive, lipid-centric explanation of microwave-mediated quality improvement in long-term stored brown rice, integrating enzyme inactivation with metabolic network reprogramming. The findings offer a novel scientific basis for applying this technology to valorize degraded grain stocks, contributing to the reduction in postharvest loss.

## 1. Introduction

The long-term storage of paddy rice often leads to irreversible quality deterioration in its milled products, representing a significant postharvest loss within the global grain supply chain [1]. This deterioration is primarily driven by the hydrolysis and oxidation of lipids in the bran layer, resulting in a marked increase in free fatty acid value (FAV), a key indicator of rancidity and a factor that downgrades rice from food-grade to feed or even industrial use [2,3]. Furthermore, rice high in free fatty acid (FFA), when used as animal feed can negatively affect animal product quality (e.g., eggs, meat), reducing both nutritional value and market competitiveness [4]. Therefore, developing effective strategies to mitigate lipid deterioration and reduce FAV in brown rice from long-term stored paddy is crucial for resource recovery and value addition.

Among non-thermal technologies, microwave treatment is a promising, rapid intervention. Its efficacy is attributed to its ability to inactivate key spoilage enzymes, such as lipase and lipoxygenase, through both thermal and potential athermal effects, thus delaying lipid hydrolysis and oxidation in grains [5]. While its effectiveness in preserving fresh grains is well-documented, there remains a knowledge gap regarding its application to already-degraded grains, specifically brown rice from long-term stored paddy [6]. Moreover, the mechanism by which microwave treatment reduces FFAs remains largely unclear. Current understanding relies primarily on bulk chemical indicators, without elucidating the underlying lipid molecular reprogramming [7,8]. This lack of mechanistic insight limits process optimization and the rational deployment of microwave technology for grain quality stabilization.

We hypothesize that microwave treatment induces a systemic remodeling of the lipid profile in brown rice from long-term stored paddy, and that this molecular-level reorganization is closely linked to the observed reduction in FFAs. To test this hypothesis and address the aforementioned knowledge gap, this study was designed to move beyond conventional quality assessment. We first optimized microwave parameters for maximal FFA reduction. Then, we employed an untargeted lipidomics approach, a powerful tool for providing detailed insights into lipid composition and metabolic pathways in food systems, including stored grains [9,10]. This approach was used to identify differential lipid species and map the altered metabolic pathways in response to treatment. Finally, integrative correlation analysis was conducted to establish links between lipidomic shifts and FFA reduction. This work provides the first mechanistic, lipid-centric explanation of microwave-mediated quality improvement in brown rice from long-term stored paddy. By integrating process optimization with advanced metabolomics, our findings offer a scientific basis for transforming microwave treatment from a generic processing tool into a targeted, knowledge-driven strategy for valorizing degraded grain stocks and improving postharvest resource efficiency.

## 2. Materials and Methods

### 2.1. Materials

*Longjing-46* brown rice samples (Heilongjiang, China) were obtained from paddy rice stored for two years (designated as fresh-stored control) and six years (designated as long-term stored) under ambient warehouse conditions (~20 °C, relative humidity ~65%). The six-year storage period exceeds the recommended duration for maintaining optimal rice quality, thus constituting a model for studying storage-induced deterioration.

### 2.2. Microwave Treatment

The brown rice samples from long-term stored paddy were subjected to microwave treatment using a laboratory microwave (JTONE-J1-3, Hangzhou, China) oven (frequency: 2450 MHz; maximum power output: 800 W). To determine the optimal processing conditions, samples were treated at two power levels: 420 W (medium-low) and 560 W (medium-high). At each power level, treatment durations of 1, 2, 3, 4, and 5 min were applied. The samples (200 g) were spread in a single layer (approximately 6–8 mm thickness) on a glass plate during treatment to ensure uniform exposure. After treatment, all samples were immediately cooled to room temperature in a desiccator prior to subsequent analysis.

### 2.3. Determination of Free Fatty Acid Value (FAV)

The FAV was determined according to a standard alkaline titration method with minor modifications [11]. Briefly, brown rice samples were ground to pass through a 425 μm sieve using an IKA M20 grinder (IKA, Shanghai, China). A precisely weighed sample (10.00 ± 0.01 g) was mixed with 50 mL of absolute ethanol in a sealed conical flask and shaken at 100 strokes/min for 10 min. After filtration, 25.0 mL of the filtrate was titrated with a standardized 0.01 M potassium hydroxide (KOH) solution using phenolphthalein as an indicator. A blank titration was performed using 25.0 mL of absolute ethanol. The moisture content of each sample was determined by oven-drying at 105 °C to constant weight. All measurements were performed in triplicate. The FAV was calculated as follows and expressed as mg KOH per 100 g of dry matter:

FAV (mg KOH/100 g dry basis) = [(V_1_ − V_0_) × C × 56.11 × 2 × 100]/(m × (1 − w)), where V_1_ and V_0_ are the KOH titration volumes (mL) for the sample and blank, respectively; C is the exact concentration of the KOH solution (mol/L); m is the sample mass (g); and w is the moisture content (g/g).

### 2.4. Fourier Transform Infrared (FT-IR) Spectroscopic Analysis

Lipid structural changes were analyzed using FT-IR spectroscopy (Nicolet iS50, Thermo Fisher Scientific, Waltham, MA, USA) with the potassium bromide (KBr) pellet method [12]. Briefly, KBr powder was dried overnight at 100 °C. Brown rice samples were finely ground and homogeneously mixed with dried KBr at a 1:100 (*w*/*w*) ratio. The mixture was pressed into transparent pellets under 8 MPa for 2 min. Spectra were acquired using a Bruker Alpha II FT-IR spectrometer (Bruker Optics GmbH, Ettlingen, Germany) in the range of 4000–400 cm^−1^ with a resolution of 4 cm^−1^. For each sample, 64 scans were co-added to improve the signal-to-noise ratio. Background spectra of pure KBr pellets were recorded and automatically subtracted from the sample spectra.

### 2.5. Lipidomics Analysis

A comprehensive, untargeted lipidomics approach was employed to profile lipid metabolites. Sample preparation followed a modified methyl tert-butyl ether (MTBE) extraction protocol [13]. Freeze-dried and finely ground (filtered through a 180 μm sieve) brown rice powder (20 mg) was extracted with 1 mL of MTBE/methanol (3:1, *v*/*v*) by vortexing for 15 min. After the addition of 300 µL of water, the mixture was centrifuged (12,000× *g*, 10 min, 4 °C). The upper organic layer was collected, dried under a gentle nitrogen stream, and reconstituted in 200 µL of methanol/isopropanol (1:1, *v*/*v*). Lipid separation was performed using ultra-performance liquid chromatography (UPLC, ExionLC™ AD, AB SCIEX, Framingham, MA, USA) equipped with a C18 column maintained at 45 °C. The mobile phase consisted of (A) acetonitrile/water (60:40, *v*/*v*) and (B) isopropanol/acetonitrile (90:10, *v*/*v*), both containing 10 mM ammonium formate and 0.1% formic acid. A 20 min linear gradient was used [14].

Mass spectrometric detection was carried out on a tandem mass spectrometer (MS/MS; QTRAP^®^ 6500+, AB SCIEX, Framingham, MA, USA) operated in both positive and negative electrospray ionization modes, essential for comprehensive lipid coverage and consistent with the UPLC-MS/MS approach reported by refs. [15,16] for lipid profiling. Prepared samples were stored at −80 °C until analysis. All operations were conducted under low-light conditions to prevent photo-oxidation. Quality control (QC) samples, prepared by pooling aliquots from all samples, were injected at regular intervals to monitor system stability.

### 2.6. Statistical Analysis

Experiments were conducted with three (for microwave treatment and FAV) or six (for lipidomics) biological replicates as specified in the respective sections. Data were expressed as mean ± standard deviation (SD). Statistical analysis was conducted using SAS 9.4 software (SAS Institute Inc., Cary, NC, USA) with one-way ANOVA and the Student–Newman–Keuls multiple-range test, with statistical significance set at *p* < 0.05. For lipidomics data, multivariate statistical analyses were performed using MetaboAnalyst 6.0 (McGill University, Montreal, QC, Canada). Unsupervised principal component analysis (PCA) was first applied to observe intrinsic clustering. Subsequently, supervised orthogonal partial least squares-discriminant analysis (OPLS-DA) was used to maximize group separation and identify lipids contributing most to the variance (VIP > 1.0). Significantly altered lipids (*p* < 0.05, fold change > 2 or <0.5) were subjected to pathway enrichment analysis based on the Kyoto Encyclopedia of Genes and Genomes (KEGG) database [17,18].

## 3. Results and Discussion

### 3.1. Optimization of Microwave Parameters for FAV Reduction

**Figure 1:**
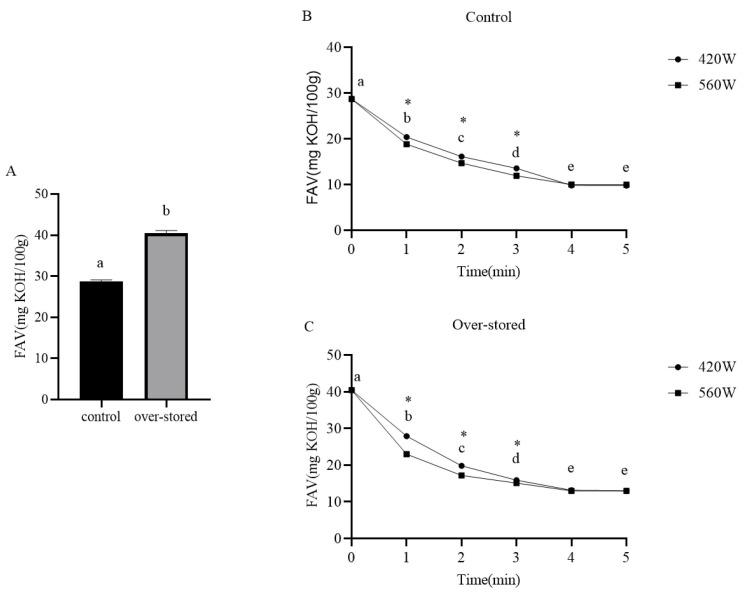
Microwave treatment reduces the fatty acid value (FAV) in brown rice milled from paddy with different storage histories. (**A**) Initial FAV of brown rice from control (2-year storage) and long-term stored (6-year storage) paddy before treatment. Asterisk (*) indicates a significant difference between the two groups (*p* < 0.05). (**B**) FAV of control brown rice after microwave treatment at 420 W and 560 W for 1–5 min. (**C**) FAV of brown rice from long-term stored paddy after the same treatments. Data are presented as mean ± SD (*n* = 3). Different letters indicate significant differences among groups with different treatment durations (*p* < 0.05).

The assessment revealed a clear effect of storage duration on lipid stability. The free fatty acid value (FAV) of the brown rice milled from long-term stored paddy (6 years) was 40.45 ± 0.69 mg KOH/100 g, significantly higher (*p* < 0.05) than that of the control (2 years) at 28.68 ± 0.42 mg KOH/100 g (Figure 1A). This 41% increase aligns with established knowledge that prolonged storage accelerates lipid hydrolysis via endogenous lipase activity, leading to free fatty acid (FFA) accumulation [19,20].

Microwave treatment proved highly effective in reversing this deterioration. As shown in Figure 1B,C, the FAV decreased significantly in a time-dependent manner at both power settings (420 W and 560 W). Statistical analysis (Table 1) confirmed that microwave power, treatment time, and their interaction all had highly significant effects on FAV reduction (*p* < 0.01). The reduction kinetics followed a biphasic pattern: a sharp, linear decrease during the first 3–4 min, after which it plateaued. Notably, the plateau FAV for long-term stored samples (~13 mg KOH/100 g) remained higher than that for control samples (~10 mg KOH/100 g), indicating a baseline level of irreversible hydrolytic damage from long-term storage. For long-term stored rice treated at 420 W, the FAV was reduced by 75.5% after 4 min, with no significant further reduction at 5 min.

A key finding was the differential response based on storage history. The maximum achievable FAV reduction rate was higher for long-term stored samples (76.3%) than for controls (69.1%) under identical conditions (560 W, 5 min). We hypothesize that this enhanced efficacy stems from the higher initial activity of lipid-degrading enzymes (e.g., lipase, lipoxygenase) in long-stored rice. Microwave treatment, through its rapid thermal effect, may more effectively denature these pre-activated enzymes, leading to a proportionally greater suppression of hydrolysis [21]. From a practical, energy-conscious perspective, treating long-term stored rice at 420 W for 4 min emerged as the optimal condition, achieving 75.5% reduction (comparable to the maximum reduction) while consuming approximately 33% less energy than the 560 W/5 min protocol.

### 3.2. Molecular-Level Evidence from FT-IR Spectroscopy

**Figure 2:**
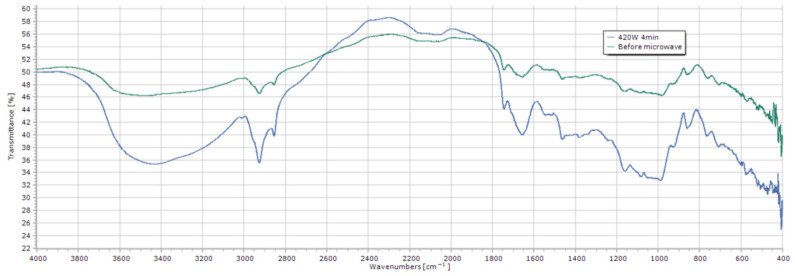
FT-IR spectra reveal molecular changes in lipids of brown rice milled from long-term stored paddy after microwave treatment. Spectra of untreated (green line) and microwave-treated (420 W, 4 min; blue line) samples are shown. Key absorption bands are assigned: 3010 cm^−1^ (=C–H stretch of unsaturated lipids), 1740 cm^−1^ (C=O stretch of esters), and 1720 cm^−1^ (C=O stretch of free fatty acids).

Fourier-transform infrared (FT-IR) spectroscopy provided direct, molecular-level evidence supporting the observed FAV trends and elucidating the structural changes in lipids. The comparative spectra of untreated and microwave-treated long-term stored rice are shown in Figure 2, with key regions annotated.

In the C–H stretching region (2800–3050 cm^−1^), the untreated sample exhibited notably weaker absorption at 3010 cm^−1^, characteristic of the =C–H stretch in unsaturated fatty acids. This attenuation is a classic indicator of oxidative scission at carbon–carbon double bonds, confirming that long-term storage induced oxidation of unsaturated lipids [22,23]. After microwave treatment, the intensity at 3010 cm^−1^ increased relative to the untreated sample, suggesting that the treatment halted or slowed further oxidation, thereby preserving the remaining unsaturated structures.

More definitive evidence came from the carbonyl stretching region (1700–1750 cm^−1^). The spectrum of untreated rice displayed a prominent peak at 1720 cm^−1^, assigned to the C=O stretch of carboxylic acids in free fatty acids (FFAs), alongside a shoulder at 1740 cm^−1^, corresponding to the ester C=O in triglycerides (TGs). The strong 1720 cm^−1^ signal visually corroborated the high FAV measurement. After microwave treatment, a decisive spectral shift occurred: the intensity at 1740 cm^−1^ increased significantly while the peak at 1720 cm^−1^ diminished. This inverse relationship provides direct spectroscopic proof that microwave treatment suppressed the hydrolysis of ester bonds in TGs, thereby reducing the accumulation of FFAs. This finding is in full agreement with the quantitative FAV data and aligns with studies on microwave mitigation of lipid hydrolysis in other cereal matrices [24].

### 3.3. Comprehensive Lipidomic Profiling and Pathway Analysis

**Figure 3:**
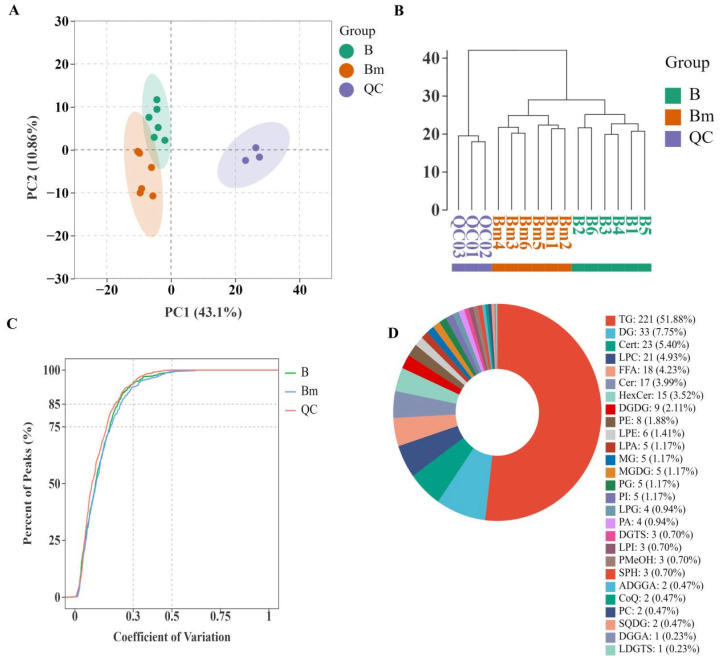
Quality control (*n* = 3) and global lipid profile of the lipidomics analysis for brown rice from long-term stored paddy (*n* = 6). (**A**) PCA score plot showing tight clustering of quality control (QC) samples. (**B**) Hierarchical clustering analysis of all samples. (**C**) Distribution of the coefficient of variation (CV) for lipids in QC samples. (**D**) Relative abundance of major lipid classes identified.

To go beyond bulk indicators and structural fingerprints, we employed untargeted lipidomics to achieve a systems-level understanding of the metabolic changes. The high quality and reproducibility of the data were affirmed by the tight clustering of quality control (QC) samples in the principal component analysis (PCA) score plot (Figure 3A) and a coefficient of variation (CV) distribution showing over 75% of lipids had a CV below 0.3 in QCs (Figure 3C), ensuring robust downstream analysis [25].

**Figure 4:**
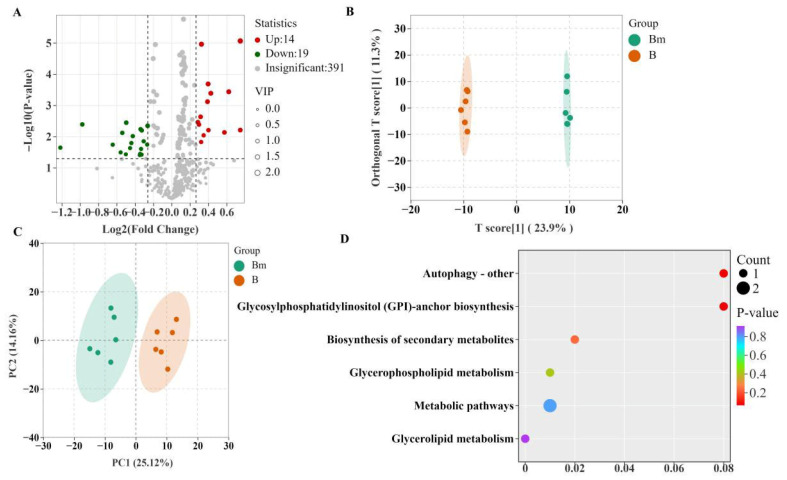
Identification of differential lipids and impacted pathways in brown rice (from long-term stored paddy) induced by microwave treatment (*n* = 6). (**A**) Volcano plot of lipids between treated and untreated groups. (**B**) PCA and (**C**) OPLS-DA score plots based on the differential lipids. (**D**) KEGG pathway enrichment analysis of the differential lipids.

A total of 27 lipid classes were identified and quantified (Figure 3D). Triglycerides (TGs), diglycerides (DGs), and ceramides (Cers) were the most abundant, reflecting the typical storage and membrane lipid composition of cereal grains [26,27]. Comparative analysis between microwave-treated and untreated long-term stored rice, using strict criteria, revealed 33 significantly differentially abundant lipids (14 upregulated, 19 downregulated; volcano plot in Figure 4A, detailed list in Table 2). Notably, among the downregulated lipids were multiple ceramide species and free fatty acids (e.g., stearic acid, heptadecanoic acid), directly linking the lipidomic shift to the observed reduction in the FAV [28]. Supervised multivariate analyses, including PCA and orthogonal partial least squares-discriminant analysis (OPLS-DA) performed specifically on these differential lipids, showed a clear and distinct separation between the two groups (Figure 4B,C), confirming that microwave treatment induces a profound reprogramming of the lipidome.

Kyoto Encyclopedia of Genes and Genomes (KEGG) pathway enrichment analysis of these differential lipids identified several significantly impacted metabolic pathways (Figure 4D). Most notably, the autophagy pathway was prominently enriched. This is a compelling finding, as autophagy is a conserved cellular process that clears and recycles damaged organelles and macromolecules, including peroxidized lipids, in response to stress. Our results suggest that microwave treatment may act as a mild stressor, activating autophagic machinery to facilitate the removal of oxidation-damaged lipids and their breakdown products, thus contributing to the overall reduction in FFA levels. This hypothesis is supported by literature linking autophagy to lipid homeostasis in eukaryotic systems [29].

## 4. Identification of Key Regulatory Lipids and Synergistic Mechanism

**Figure 5:**
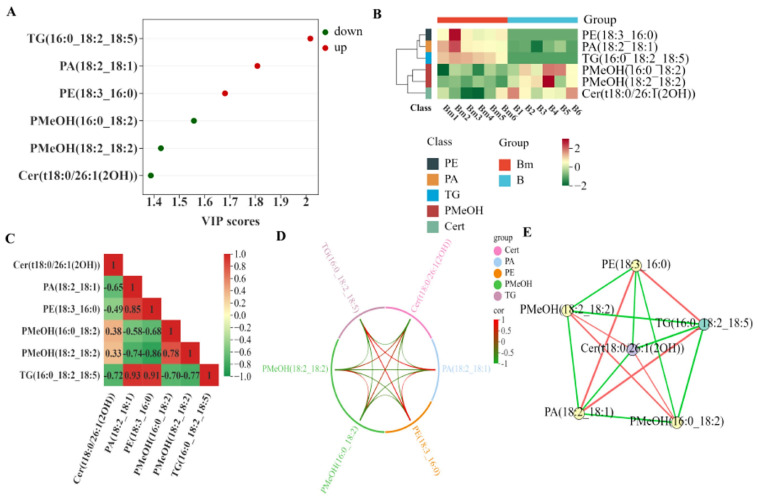
Identification and correlation analysis of key lipids associated with FAV reduction in microwave-treated brown rice from long-term stored paddy (*n* = 6). (**A**) VIP scores from the OPLS-DA model. (**B**) Hierarchical clustering heatmap of key lipids. (**C**) Correlation heatmap, (**D**) chord diagram, and (**E**) network analysis depicting correlations among key lipids.

To extract actionable insights from the complex lipidomic data, we identified the lipids most critical for distinguishing the treated and untreated states using Variable Importance in Projection (VIP) analysis from the OPLS-DA model (Figure 5A). Three lipids with VIP scores > 1.5 were highlighted as key contributors: two phosphatidyl methanol species (PMeOH(16:0_18:2) and PMeOH(18:2_18:2)) and a hydroxylated ceramide (Cer(t18:0/26:1(2OH))). All three were significantly downregulated following microwave treatment (Table 2), with PMeOH(18:2_18:2) showing the most dramatic decrease (fold change = 0.43).

Subsequent correlation analyses, including a correlation heatmap (Figure 5C), a chord diagram (Figure 5D), and a network analysis (Figure 5E), revealed a strong positive correlation among these three key lipids. This indicates their levels co-vary, suggesting they are metabolically linked and undergo coordinated, synergistic degradation in response to microwave energy. The degradation of these specific lipids presents a novel mechanistic insight. While triglyceride (TG) hydrolysis directly contributes to FFAs, the downregulation of phosphatidyl methanol (PMeOH), a less common glycerophospholipid, and ceramide (Cer), a central sphingolipid involved in signaling, suggests broader regulatory effects. The hierarchical clustering heatmap (Figure 5B) further visually corroborates that these key lipids cluster together and exhibit a consistent downregulation pattern in the treated group. Ceramides are known to interact with fatty acid metabolism, and their alteration can influence overall lipid homeostasis [30]. Microwave treatment, through its combined thermal and potential non-thermal field effects, may simultaneously inactivate hydrolytic enzymes and disrupt the metabolic network connecting these lipid pools.

## 5. Conclusions

This study demonstrates that microwave treatment is an effective technology for rapidly improving the lipid quality of brown rice derived from long-term stored paddy, addressing the challenge of postharvest grain loss. The optimal treatment condition (420 W, 4 min) reduced the fatty acid value by 75.5%, achieving a balance between efficacy and energy efficiency. Beyond direct enzyme inactivation, the novel mechanism involves a microwave-induced reprogramming of the lipid metabolic network. This reprogramming is characterized by the synergistic downregulation of key lipid species, including specific phosphatidyl methanols and ceramides, and the potential activation of the autophagy pathway, collectively mitigating lipid hydrolysis and oxidation.

These findings provide the first mechanistic, lipid-centric elucidation for microwave-mediated quality restoration in long-term stored grains, moving beyond phenomenological description. They establish a scientific foundation for applying microwave technology in the valorization of degraded grain stocks for feed use. Future research should focus on scaling up the process, conducting feeding trials to validate nutritional outcomes, and exploring the economic feasibility of industrial implementation to bridge the gap between laboratory proof-of-concept and practical application in sustainable postharvest management.

## Figures and Tables

**Table 1:** Effects of microwave power and treatment time on the fatty acid value (FAV) of brown rice from paddy stored for different durations.

Time	Treatment Methods	Treatment Time (min)	Moisture Content (%)		FAV (mg KOH/100 g)
long-term stored	Before microwave		10.76 ± 0.77 ^a^		40.45 ± 0.69 ^a^
	420 W	1	10.12 ± 0.10 ^b^		27.88 ± 0.36 ^c^
		2	9.34 ± 0.11 ^cd^		19.80 ± 0.02 ^f^
		3	8.18 ± 0.14 ^e^		15.89 ± 0.02 ^i^
		4	7.44 ± 0.37 ^f^		13.13 ± 0.30 ^l^
		5	6.6 ± 0.33 ^g^		13.01 ± 0.30 ^l^
	560 W	1	9.62 ± 0.11 ^c^		22.97 ± 0.03 ^d^
		2	8.60 ± 0.13 ^e^		17.19 ± 0.03 ^h^
		3	7.26 ± 0.07 ^f^		15.12 ± 0.02 ^j^
		4	6.32 ± 0.12 ^g^		12.97 ± 0.36 ^l^
		5	6.25 ± 0.17 ^g^		12.97 ± 0.33 ^l^
control	Before microwave		9.37 ± 0.61 ^cd^		28.68 ± 0.42 ^b^
	420 W	1	9.12 ± 0.09 ^d^		20.37 ± 0.02 ^e^
		2	8.31 ± 0.12 ^e^		16.11 ± 0.33 ^i^
		3	7.51 ± 0.10 ^f^		13.55 ± 0.34 ^k^
		4	6.61 ± 0.45 ^g^		9.81 ± 0.30 ^n^
		5	6.43 ± 0.36 ^g^		9.80 ± 0.31 ^n^
	560 W	1	8.57 ± 0.15 ^e^		18.82 ± 0.36 ^g^
		2	8.28 ± 0.25 ^e^		14.68 ± 0.04 ^j^
		3	7.39 ± 0.11 ^f^		11.91 ± 0.36 ^m^
		4	6.59 ± 0.18 ^g^		10.01 ± 0.33 ^n^
		5	6.30 ± 0.07 ^g^		9.98 ± 0.34 ^n^
*p*	*p* time			<0.01	
	*p* treatment methods			<0.01	
	*p* time × treatment methods			<0.01	

Note: Values are expressed as mean ± standard deviation (*n* = 3). Different superscript lowercase letters within a column indicate significant differences (*p* < 0.05).

**Table 2:** Significantly differential lipids identified in brown rice (milled form long-term stored paddy) after microwave treatment compared to the untreated control.

Material	Classification	FC	*p*-Value	Type
Ceramide_Cer(d18:1/22:0)	Sphingolipids(SP)	0.8	0.01	down
Ceramide_Cer(t17:0/24:0)	Sphingolipids(SP)	0.74	0.01	down
Ceramide_Cer(t18:0/20:0(2OH))	Sphingolipids(SP)	0.83	0.02	down
Ceramide_Cer(t18:0/26:1(2OH))	Sphingolipids(SP)	0.64	0.02	down
Ceramide_Cer(t18:1/26:0)	Sphingolipids(SP)	0.74	0.02	down
Coenzyme Q10(oxidizing type)	Isoprenol lipids(PR)	0.79	0.01	down
Diacylglycerol_DG(18:2_18:3)	Glycerides(GL)	1.32	0	up
Diacylglycerol_DG(18:2_20:1)	Glycerides(GL)	1.23	0	up
Diacylglycerol_DG(18:1_18:2)	Glycerides(GL)	1.34	0	up
Diacylglycerol_DG(16:0_18:3)	Glycerides(GL)	1.22	0	up
Diacylglycerol_DG(18:1_18:1)	Glycerides(GL)	1.25	0	up
Diacylglycerol_DG(16:0_18:2)	Glycerides(GL)	1.24	0	up
Diacylglycerol_DG(18:1_24:0)	Glycerides(GL)	1.49	0.01	up
Diacylglycerol_DG(18:1_20:0)	Glycerides(GL)	1.25	0.01	up
Diacylglycerol_DG(16:0_18:1)	Glycerides(GL)	1.31	0	up
Octadecanoic acid	Fatty acyl groups(FA)	0.79	0.02	down
Octadecanoic acid	Fatty acyl groups(FA)	0.81	0.01	down
Stearic acid	Fatty acyl groups(FA)	0.73	0.02	down
Heptadecanoic acid	Fatty acyl groups(FA)	0.68	0.03	down
Erucic acid	Fatty acyl groups(FA)	0.79	0.04	down
Lysophosphatidylethanolamine_LPE(18:0)	Glycerophosphatides(GP)	0.83	0	down
Lysophosphatidylethanolamine_LPE(18:3)	Glycerophosphatides(GP)	0.8	0.04	down
Phosphatidylic acid_PA(18:2_18:1)	Glycerophosphatides(GP)	1.54	0	up
Phosphatidylic acid_PA(18:2_18:2)	Glycerophosphatides(GP)	1.32	0.01	up
Phosphatidylethanolamine_PE(18:2_16:0)	Glycerophosphatides(GP)	0.71	0	down
Phosphatidylethanolamine_PE(18:3_16:0)	Glycerophosphatides(GP)	/	0.01	up
Phosphatidylethanolamine_PE(18:1_18:2)	Glycerophosphatides(GP)	0.71	0.04	down
Phosphatidylethanolamine_PE(18:2_18:2)	Glycerophosphatides(GP)	0.69	0.01	down
Phosphatidyl methanol_PMeOH(16:0_18:2)	Glycerophosphatides(GP)	0.51	0	down
Phosphatidyl methanol_PMeOH(18:2_18:2)	Glycerophosphatides(GP)	0.43	0.02	down
Triglyceride_TG(20:1_20:1_18:2)	Glycerides(GL)	0.78	0.04	down
Triglyceride_TG(16:0_18:2_18:5)	Glycerides(GL)	/	0	up
Triglyceride_TG(18:1_18:3_22:1)	Glycerides(GL)	1.27	0.01	up

Note: Lipids were filtered with criteria of VIP > 1.0, fold change > 2 or <0.5, and *p*-value < 0.05 (*n* = 6).

## Data Availability

The original contributions presented in this study are included in the article. Further inquiries can be directed to the corresponding author.
